# News from Africa: Novel Anopheline Species Transmit *Plasmodium* in Western Kenya

**DOI:** 10.4269/ajtmh.16-0020

**Published:** 2016-02-03

**Authors:** Jan E. Conn

**Affiliations:** Wadsworth Center, New York State Department of Health, Albany, New York; Department of Biomedical Sciences, School of Public Health, University at Albany, State University of New York, Albany, New York

Multiple species masquerading under a single species name—that is, cryptic species that are morphologically indistinguishable—are remarkably widespread, from red algae, through tapeworms infecting humans, to bats and fish. Their detection is of utmost importance in understanding diversity at local and regional levels^1^; conservation biology depends in part on evaluation of community diversity, which in turn frequently requires molecular plus morphological taxonomy for accurate identification of the species comprising a given community. To those involved in public health, the importance of accuracy cannot be understated, because not all members of mosquito species complexes are equal in susceptibility to pathogens or in their ecology, biology, or abiotic requirements.^2^ In particular, the discovery of previously undescribed cryptic anopheline species has become more common, although there can be occasional discrepancies when studies rely exclusively on the mitochondrial DNA barcode sequence.^3^ Of the 465 formally named anopheline species worldwide,^4^ about 41 (9%) have been incriminated as malaria vectors.^5^

Thus, the detection of new potential malaria vectors in Kenya has several important ramifications. The study “Molecular characterization reveals diverse and unknown malaria vectors in the western Kenyan highlands” by St. Laurent and others, published in this issue, is a welcome addition to our understanding of African regional biodiversity, adds to the anopheline database represented by internal transcribed spacer 2 (*ITS2*) and cytochrome c oxidase subunit 1 (*COI*) GenBank sequences, and is of significance to global efforts toward malaria control and elimination.^6^ This publication expands upon a letter that described newly identified outdoor, early-biting, potential anopheline vector species from the western highlands of Kenya^7^ by including adult and larval collections from villages in Nyanza Province. These two studies and several others are part of a larger project, the Malaria Transmission Consortium studies in the Kenya highlands.^8^

Excitingly, another recent article that includes many of the same authors also describes new malaria vector species from eastern Zambia and several of their associated bionomics features.^9^ Together, these articles add to our knowledge base of African anopheline species. More importantly, they are informative in relation to malaria interventions, because new data on accurately identified mosquito species, their insecticide resistance status, host preference, and vector or potential vector infectivity rates, will help to explain aspects of the persistence of malaria despite high intervention coverage in some regions.^10^–^12^

The most prevalent species from western Kenya in the new report were, as expected, *Anopheles funestus* s.l. and *Anopheles arabiensis*, the latter which is the third most abundant species and was not infected with *Plasmodium*. On the basis of a phenogram created by Bayesian analysis of *ITS2* sequences from representatives of all species collected, the authors detected nine groups that are either new species or correspond to morphologically named species for which either *ITS2* or *COI* sequences are unavailable for comparison. These are named A, F, G, I, K, N, O, P, and Q. Three, A, F, and G, were infected with *Plasmodium falciparum*, which was detected by using enzyme-linked immunosorbent assay and confirmed by polymerase chain reaction (PCR); I (identified by *ITS2* as *Anopheles theileri*) was infected with *Plasmodium*, which was only detected with PCR. A, identified morphologically as a member of the *Anopheles demeilloni* group, is of considerable interest because it was relatively abundant and its infectivity rate was similar to that of *An. funestus* in this and other studies in western Kenya.^13^,^14^ Group A was also detected in Zambia,^9^ suggesting it may be of regional rather than local importance in malaria transmission. Although F and G were not very abundant, their infectivity with *P. falciparum* supports further investigation. *Anopheles leesoni*, previously identified in western Kenya, Tanzania, and west Africa, was provisionally identified in the new report (based on *ITS2* sequences only) and was shown to be infected with *Plasmodium* for the first time.

Groups K, N, and P were identified to subgenus *Cellia*, either Cellia or Myzomyia series; whereas O and P were subgenus *Anopheles*. None of these five groups was abundant; nonetheless every effort should be made to accurately identify them, as they may be members of known complexes. Small sample sizes preclude conclusions about potential involvement of these groups in malaria transmission.

Although the impetus to collect and identify additional anophelines in western Kenya stems from a desire to improve malaria transmission evaluation tools and control,^15^ this characterization is likely to stimulate additional regional studies that have great potential to better characterize anopheline species, whether they are currently involved in malaria transmission.
